# Review of Brain-Machine Interfaces Used in Neural Prosthetics with New Perspective on Somatosensory Feedback through Method of Signal Breakdown

**DOI:** 10.1155/2016/8956432

**Published:** 2016-05-30

**Authors:** Gabriel W. Vattendahl Vidal, Mathew L. Rynes, Zachary Kelliher, Shikha Jain Goodwin

**Affiliations:** Department of Biomedical Engineering, University of Minnesota, Minneapolis, MN 55455, USA

## Abstract

The brain-machine interface (BMI) used in neural prosthetics involves recording signals from neuron populations, decoding those signals using mathematical modeling algorithms, and translating the intended action into physical limb movement. Recently, somatosensory feedback has become the focus of many research groups given its ability in increased neural control by the patient and to provide a more natural sensation for the prosthetics. This process involves recording data from force sensitive locations on the prosthetics and encoding these signals to be sent to the brain in the form of electrical stimulation. Tactile sensation has been achieved through peripheral nerve stimulation and direct stimulation of the somatosensory cortex using intracortical microstimulation (ICMS). The initial focus of this paper is to review these principles and link them to modern day applications such as restoring limb use to those who lack such control. With regard to how far the research has come, a new perspective for the signal breakdown concludes the paper, offering ideas for more real somatosensory feedback using ICMS to stimulate particular sensations by differentiating touch sensors and filtering data based on unique frequencies.

## 1. Introduction

Limb loss and paralysis can have crippling consequences for those affected, severely hindering the ability of individuals to live normal lives. Work being done with prosthetics utilizing neural interfacing techniques for neural control may hold the answers to potentially restoring some of what these people have lost and significantly improving their overall quality of life [1, 2]. With prosthetics that can effectively utilize a brain-machine interface (BMI), patients can control computer cursors to animatronic limbs using signals recorded and decoded by their brain [2, 3]. Research is currently focused on refining these techniques and incorporating different forms of sensory feedback for increased control [2, 4].

Recently the importance of somatosensory feedback as a method for providing higher level cognitive control has become apparent [5–7]. Ever since animal studies showed the efficacy of decoding neural signals and translating them into physical limb movement [1, 8], research has been done in conveying tactile information by stimulating both peripheral nerve bundles [9] and the somatosensory centers [10] of the brain directly, leading to an increase in coordination and limb control. Leading and innovative technology, focused around BMIs that can both encode and decode information, is now entering its clinical phase, making it a subject worthy of in-depth discussion and speculation.

This paper will review what is currently being done with prosthetics implementing BMI technology, focusing on the integration of proprioceptive and somatosensory feedback as a method to improve control. After delving into some of the basics behind BMI, the importance of sensory feedback and commencing with the review, a new perspective on methods for signal breakdown will be explored for the purpose of offering possible new insights and a unique perspective on what can be done moving forward in the field. Finally, the applications of neural prosthetics will be discussed.

## 2. Brain-Machine Interfaces

A brain-machine interface is a link between the mind and the physical world in which information can flow and allow the two to interact through an external device. The principals behind modern day prosthetic BMIs involve extracting motor control signals from pools of neurons and translating those signals into motor control of a device that control being fine-tuned through different feedback sources and assistance from computer algorithms [11]. Information flow is governed by the ability to record signals from neurons and decode those signals so that they can be translated into device control; information can also be encoded and sent back into the brain through electrical stimulation techniques [5, 11].

### 2.1. Neural Decoding and Interfacing

**Figure 1:**
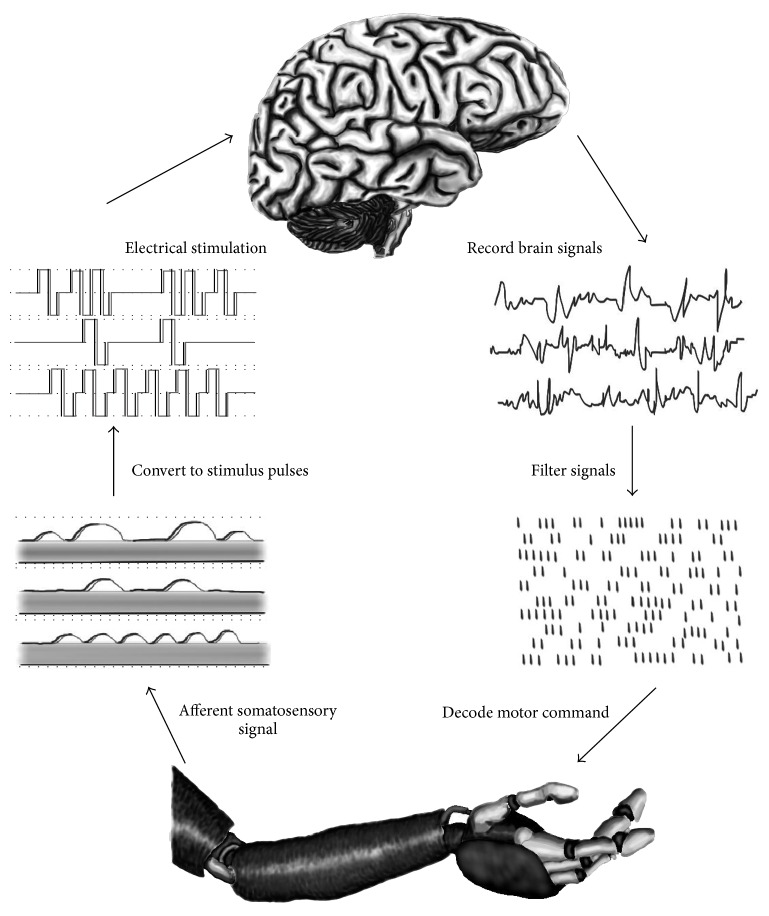
Working of neural prosthetics using a brain-machine interface. Afferent somatosensory signal is taken from the prosthetic device and is fed into the brain, from where the motor signal is sent back to the prosthetic limb [5].

Decoding is a huge component of being able to understand brain signals and translating them to the prosthetic device. Decoders use multiple mathematical functions to process a variety of neural signals recorded by electrodes into signal patterns that can be recognized and interpreted as relating to specific neural activity [14]. The right side of Figure 1 depicts this decoding process. Recording of these signals can be both invasive and noninvasive. Invasive methods span from recording a series of action potentials, to the activity of a small group of neurons, large neuronal ensembles, or local field potentials (LFPs) [11]. EEG signals are recorded from the surface of the head and decoded in noninvasive BMIs. Probability distributions are generated which can predict the meaning behind collected data, and lines are drawn between the significance of one response in comparison with several others in the form of tuning curves [15, 16]. Given the probability of specific firing patterns corresponding to intended movement, an external device can be controlled mentally if the mutual information recorded can be correlated with that intent.

Along with decoding, one of the most crucial challenges facing brain-machine interfacing is electrode design and lifespan. The electrode/electrolyte interface can cause unfavorable reactions, such as irreversible faradaic reactions, and chronic implantation in the central nervous system has been shown to elicit a typical response of electrode encapsulation by macrophages, microglia, and astrocytes, even in materials considered to be biocompatible [18]. Less invasive methods such as EEG improve upon eliminating these unfavorable reactions, but at the cost of special and temporal resolution, which often suffer given the overlap of electrical activity in other cortical areas; noise can also increase given the different tissues the signals have to traverse [11].

### 2.2. Cognitive Control

Decoding signals from neural activity collected from these electrodes give rise to cognitive control. BMIs used in research involving monkeys have developed from giving them cognitive control of a cursor on a computer screen, in which they would perform tasks for a juice reward [15], to being able to control animatronic limbs to multiple degrees of freedom, allowing the monkeys to feed themselves through neural control of the prosthetics [19].

Patients suffering from paralysis and limb loss can theoretically benefit from two different types of prosthetics: motor-based prosthetics that read out information from motor cortical areas of the brain concerned with arm and movement and cognitive prosthetics that translate activity from various cortices related to sensory-motor integration involving higher level of cognitive processes that organize behavior [19, 20]. Efferent interfaces also rely on decoders that implement biomimicry, defined as natural mapping between brain activity and limb movement, or a corresponding method that attempts to produce natural patterns to control the state of a limb [5]. The recorded neural activity is decoded based on a chosen mathematical model correlating specific activity with intended movement, which translates into the motions produced by the prosthetics. However, even these types of BMIs are limited since they only include efferent signals sent from the brain to the device, meaning feedback is limited to the ability of the operator to see the device while in action. An afferent interface related to somatosensory feedback could be the answer to improving these BMIs, and attention is now being turned to encoding information in the form of somatosensory stimulation, which has its value and implications concerning prosthetic BMI limb control [16].

## 3. Enhanced BMI with Sensory Feedback

The idea of sending the information about the touch from the artificial hand to the brain is a new concept. It works by adding additional somatosensory feedback channel that can create tactile signals generated by real sensors placed in the robotic hand, directly to the somatosensory cortex. The goal of adding sensory signal makes the system closer to the real side and can also add a whole new experience of being able to feel the touch surface. BMI without somatosensory feedback only relies on visual feedback and thus could result in reduced quality of BMI-controlled movements [4, 5].

### 3.1. Proprioception and Improved Control

Research has shown that integration of multiple sources of feedback significantly improves control when using BMIs, research that will be explored in the following animal study. Work done in 2010 by Suminski et al. using BMIs that incorporated feedback from multiple sensory modalities in monkeys found early on that using proprioception as a feedback mechanism aided in neural control [2].

**Figure 2:**
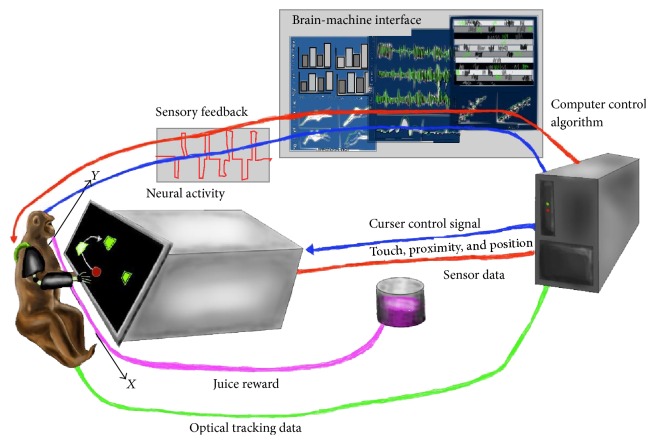
Illustration that depicts the set-up using an exoskeleton to incorporate proprioception into the mind controlled computer curser operated by the monkey [2].

Two adult male rhesus macaques were trained to control a two-dimensional cursor using a robotic exoskeleton, moving the cursor to a series of random targets to receive a juice reward. The monkeys were then implanted with a 100-electrode microelectrode array in the primary motor cortex (MI) contralateral to the arm used for the task of controlling the cursor (see Figure 2), and multiple forms of feedback were compared regarding the ability to control the arm. Visual feedback only involved the money moving the cursor without moving its arm, but the visual and proprioceptive feedback condition involved the monkeys arm being moved by the exoskeleton to follow the visual cursor. A real time decoder, based on a linear filter, was implemented using a Wiener filter, and a time series of hand position data points was reconstructed from a linear combination of neural responses from many neurons at multiple times. Examination of the cursor trajectories in each condition showed that the BMI incorporating both vertical visual and proprioceptive feedback was faster and straighter compared with visual feedback alone. Information was recorded from spiking neural activity, and it was found that mutual information often peaked at positive lags, indicating cell activity was carrying information about the future state of the cursor; but given the addition of proprioception, some neurons peaking mutual information occurred with no or negative time lag, suggesting a sensory-type response. Overall, mutual information about cursor movement was strongest (125% increase) during the condition where monkeys had both visual and proprioceptive feedback about the decoded cursor movement when compared to just visual feedback [2].

These results are the first to demonstrate the important implications of feedback modalities other than that provided by one's vision in cortically controlled brain-machine interface. The performance with proprioception also surpassed that reported in a clinical experiment involving two human patients with tetraplegia [21] and compared favorably with the state-of-the-art BMIs that rely on vision for closed loop control. The mutual information analysis provided the strongest evidence of the improvement of BMI control being a result of proprioceptive feedback. Improved BMI performance due to sensory input could have been the result of visual and kinesthetic feedback providing a more accurate estimate of the state of the system, or because the kinesthetic feedback generated by moving the arm was likely smoothed with respect to the visual feedback due to the dynamics of the arm/exoskeleton [2].

### 3.2. ICMS and Somatosensory Restoration

Somatosensation, including proprioception, is an integral component of natural motor abilities. Losing proprioception will have significant detriments on the capacity to plan dynamic limb movements [22], and in previous experiments, S1 lesions in monkeys led to uncoordinated finger movements [23]. Researchers have been attempting to employ biomimicry to convey sensory feedback as well, using intracortical microstimulation (ICMS) pulse trains carried through implanted electrodes in monkeys [5]. However, it is currently impossible for wide scale neuron activation to occur with the specificity needed to evoke a particularly detailed desired response. To discuss the restoration of somatosensation via ICMS, this paper will mostly focus on using stimulation strategies to mimic closely the cortical activity caused by contact with a native limb. This involves restoring different aspects of touch and proprioception on the foundation of an understanding of the natural coding in S1 and relevant cortical areas. Much of the research confirming the viability of this approach has already been done including Wilder and Penfield's cortical stimulation experiments in the 1930s and 1940s [24].

Conveying somatosensory information via ICMS has also been shown to be viable in animal studies. Researchers at the University of California Berkeley tested whether rats could use artificial tactile percepts generated from ICMS to the barrel cortex to navigate around a virtual target [25]. Rats were implanted with microwires, a type of microelectrode array, in the infragranular layer of barrel cortex. The rats were first trained to detect ICMS, which elicited sensory percepts, and then one whisker of the rat was tracked in real time using a light foam marker. The rat was then placed on target localization trials, where it would first encounter a real object and use it to locate a target for a reward, and then the target was removed and replaced with ICMS to the barrel cortex to define a virtual location. In this experiment, the rat was able to locate the virtual targets placed around the rat with much greater accuracy than chance. A separate experiment was also performed where rats had to detect ICMS pulses over a variable time interval. The rat was able to distinguish between distractor mechanical stimuli and ICMS pulses to replicate the target stimulus to receive a reward. These experiments demonstrate the viability of ICMS as a method of generating useful somatosensory percepts for animals. In addition to somatosensory feedback delivered to simulate a virtual object, ICMS have also been used to convey localized tactile percepts with natural features [25].

Researchers at the University of Chicago sought to convey information about sensory contact location on the hand and digits of a Macaque monkey by delivering ICMS to regions of S1 with particular receptive fields in the hand [26]. Tabot and colleagues first placed a microelectrode array into the area of S1 known to have RFs for the hand, and then they localized the RFs for each finger by applying a mechanical stimulus and recording LFPs via the microelectrode array. They used this information to present information conveying mechanical stimulus of varying pressure via ICMS with results comparable to native fingers. From this, it was concluded that stimulation could be used to mimic cortical responses to tactile contact events [26].

Early experiments exploring sensory mapping along S1 via electrical stimulation by Penfield and Boldrey showed that S1 neurons organized into distinct columns, which represent regions of the body [24], and that electrical stimulation of the neurons could convey sensory information dependent on the region of S1 stimulated [27]. Twenty years later, Mountcastle suggested that neurons which respond to similar stimuli are organized into functional columns along S1 [28]. Many later studies found evidence of columns in S1 encoding sensory information for individual digits of the hand and an even higher level of modular organization of the particular types of receptors within each digit [29, 30]. The idea of this paper is to utilize these findings and advancements in the field of neuroscience as a foundation to improve encoding of somatosensory feedback for somatosensory BMIs.

One option being explored utilizes the brains ability to adapt, relying on its plasticity, instead of meeting the demands of complete biomimicry. By associating different stimulation patterns with various kinds of sensory information, it is hoped that patients will be able to learn the meaning behind each and use that information to operate better prosthetics. This method might also be warranted in the presence of cortical plasticity, or when the brain adapts unused areas to help control the functions used to compensate for that loss. Given time, areas not in use may have become completely devout to this other function, making new adoption out to be a more reliable option. The synergy between these adaptive methods and biomimicry may hold answers to improving patient control of cognitive neural prosthetics and BMIs that can integrate both afferent and efferent signals [5].

## 4. Applications

### 4.1. Clinical Applications

BMI technology can aid tremendously in restoring function and patient rehabilitation in a clinical setting. Traditionally, the main forms of treatment for sensorimotor disorders involve pharmaceutical intervention for pain and clonus, and classic physiotherapy techniques focused on avoiding muscular hypotrophy by administration of passive movement sessions through manual interventions [31]. However robotic-assisted training has increased in clinical settings, an example of this being the semiexoskeletal robot ARMin II [32], and is sometimes coupled with virtual reality retraining programs, which can help integrate visual, auditory, and tactile stimulation [33]. With the advent of BMI technology, it suddenly became possible to combat traumatic or degenerative sensorimotor impairment given its restorative applications. Devices that send signals to the brain and decode from the brain allow for prosthetics that can provide sensory input and receive commands for interaction with the environment. Sensory inputs can range from visual, sound, and somatosensory feedback, and cognitive neural prosthetics account for the pinnacle of complex and flexible mind control device.

The idea of neural prosthetic control through functional electrical stimulation spans back from when one of the first FES systems employing implantable electrodes is inserted into muscular fibers to allow a hemiplegic patient to move a completely paralyzed limb again [34] to FES-BCI joint approaches demonstrating that noninvasive solutions for restoring lost motor functions can be as effective as invasive procedures. FES sensory feedback could help decoding of intended movement, enhancing patients performances. Limitations on noninvasive stimulation include low selectivity in muscular stimulation, weakness in deep muscles activation, difficulty in movement repeatability, and pain. Limitations of invasive methods include risks of infection, rejection, neural plasticity, and cellular death [7].

Implanted electrode arrays can produce excellent accuracy and complex motor routines; modern BMIs can detect and encode natural hand and finger motions performed by monkeys with the use of intracortical electrodes [35, 36]. Intracortical electrode use is limited in humans, but ECoG-based BMIs have proven to be reliable. The brain is able to balance reciprocally the incoming sensory information and the outgoing motor command through inverse and forward internal predictions of the expected motor outcome and the associated sensory consequence [7]. Inverse kinematics, using kinematic equations to approximate the parameters needed for the robotic device to reach a specific state, are being looked at to compensate for the mostly nonlinear relationship between sensory and motor information.

Raspopovic et al. at Ecole Polytechnique Federale de Lausanne set out to restore natural sensory feedback via stimulation of peripheral sensory nerves (median and ulnar nerves) through the use of transversal intrafascicular multichannel electrodes [37]. They then tested whether this information could recover near-natural sensation that the patient could use to identify several features of objects they touched while blindfolded and acoustically shielded. They found that the artificial sensory feedback alone allowed the patient to accurately control applied force to avoid crushing objects in the absence of other feedback (visual or auditory). In their first experiments, stimulation current varied directly with the reading of the sensor attached to the finger of the prosthetic limb. Similar experiments using nerve stump signals utilized EEG-driven analysis of peripheral neural signals during amputee patient training to improve motor command classification [38]. Later, Oddo and colleagues in the same group used a real time model of the Izhikevich spiking neuron to generate the stimulation current [39, 40]. This vast level of control is what gives neural prosthetics with BMIs that can convey tactile information the advantage over those without. Improving the signal can further increase control and make the feedback seem more naturalistic.

Future perspectives include incorporating the prosthesis into the body schema (summing all of the sensory information). Better brain-prosthetics integration must reduce the timing between motor information and sensory information, which has been found to be crucial for patient recognition and acceptance of the prosthetics [41]. Existing invasive methods have demonstrated the capability of restoring this timing [26, 37]. Furthermore, compacting the computational technology for portability must be accomplished. This will become critical as signal-processing techniques develop further, possibly enabling the improvement of decoding and encoding signals.

### 4.2. Peripheral Nerve Stimulation

**Figure 3:**
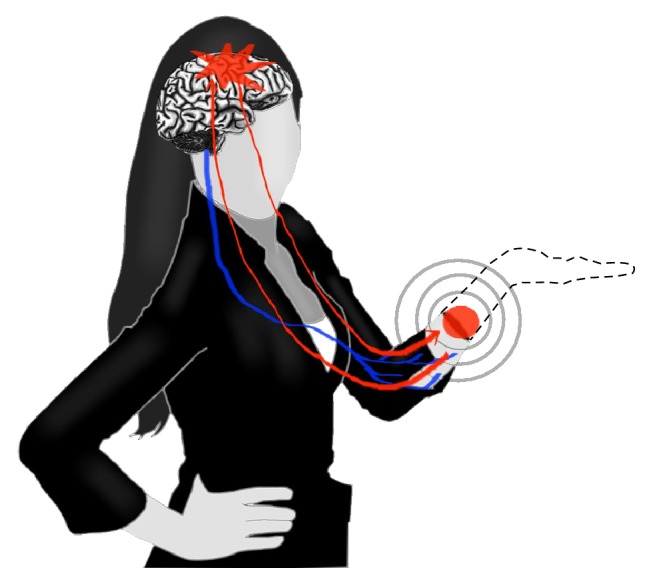
Phantom limb pain depiction. The nerve endings (located at the red circle), still present at the site of the amputation, send signals (red arrows) or the cortical reorganization (red star in the brain) generates the phantom limp pain [17]. Other sensations that can be felt involve tingling, cramping, heat, and cold.

Dealing with phantom limb pain as an application of ICMS offers a good example of the mechanisms and benefits of peripheral stimulation in the context of neural prosthetic replacement. Phantom limb pain is a mild to extreme pain whose seeming origin comes from a no longer existing extremity or which is felt from where a person's limb has been amputated [17, 42]. This pain might exist partly because of the still intact nerve endings at the site of the amputation, or because of harmful cortical reorganization [17, 42, 43].  Figure 3 diagrams phantom limb pain, including the nerve endings and signals that are being sent back and forth between the limb and brain. The sensory information being sent to the brain will also create the illusion that the limb is still there.

Even when a basic prosthetic device is attached to an upper limb injury the patient can still have these types of phantom pains because there is still no sensory information being sent to the primary somatosensory area of the brain. The sensory information received at the prosthetic device has shown a resultant decrease in the phantom pain experienced by these amputee patients [9, 44]. Reducing this phantom pain has value as a stand-alone application of prosthetics offering ICMS for touch information. However its uses toward treating issues of phantom limb do not end there. It has been demonstrated that tactile perception can be recreated with neural interfaces with these peripheral nerves [45]. This natural touch perception can help a person feel that their prosthetics is more of a replacement arm than a machine, allowing for a functional restoration unlike any previous treatment could offer. It is easier to overlook or underestimate the importance of having something that feels and acts like a normal hand in favor of a device whose objective is to simply perform a desired task as seen from the perspective of the patient. Being able to feel and grasp an object is very important when using a prosthetic device, but what might be the most important aspect is simply being able to feel another person. Restoring these feelings to people should be the main drive for adding sensory feedback information and, more importantly, the touch perception [12, 26, 41, 45].

### 4.3. Other Forms of Stimulation

Although most of the current research involves the use of ICMS to stimulate the somatosensory cortex; in future, the use of optogenetics might become more prevalent. Optogenetics is based on genetically modified ion channels that respond to light, and thus it could remove all the problems associated with the use of ICMS. It would also allow for finer control of spatial pattern of activation [46].

## 5. New Perspectives

The focus on improving control in this paper will be via proposing a stimulation method to provide full sensory information directly to the patient. The intent on conveying comprehensive somatosensory information will focus on providing information regarding multiple features of tactile perception rather than just one (e.g., force and vibration frequency rather than just one of them). Visual feedback has been predominantly used to modulate the output of BMI limbs, but this provides limited control of force and takes time for complicated spatial tasks [22]. As mentioned earlier, the insufficiency of visual feedback for complex tasks was addressed by Raspopovic and colleagues in a successful attempt at using microelectrodes to convey tactile percepts to the patient [37]. In their paradigm, the information was conveyed via stimulation of peripheral nerves, and the stimulation current varied directly with the reading of the sensor attached to the finger of the prosthetic limb. Though peripheral stimulation was adequate in eliciting tactile perception related to force, the use of ICMS may open the possibility of conveying multiple types of somatosensory information to the patient [8].

Current ICMS techniques have demonstrated the capability to deliver localized activation of neurons [47]. They can be used to convey successfully variable somatosensory percepts that monkeys can discriminate [48], and the tactile percepts are useful enough to aid in the completion of spatial tasks in monkeys [49]. ICMS have also been used to elicit fine motor movements in monkeys to aid in cortical mapping [23].

As elucidated earlier, there has been much progress in the development of advanced decoding algorithms and research into the function and mapping of the motor cortex that has contributed to the capability of lending CNP arms to mimic much of the natural movement capabilities of a native arm. Given these developments and the demonstrable advantage of artificial sensory feedback [50], using the direct ICMS to S1 could allow for superior feedback than exclusively visual feedback to be conveyed to the patient.

Since ICMS has been established as the preferred method of conveying somatosensory feedback, it is necessary to have a stimulation strategy that will successfully convey comprehensive somatosensory information. To accomplish this, the signals will first have to be acquired by the device and then converted into a form that can be conveyed via ICMS. The following proposed method of signal breakdown will only require information from one or two sensors preferably from a flexible array sensor with a high spatial resolution and small size accompanied by a larger normal force sensor to contrast related receptive fields in the hand. This combination of sensors is a possibility given previous applications in robotics [51].

### 5.1. Method of Signal Breakdown

Next, the method of signal breakdown will be discussed. Since most of the information within contact events is contained within the event itself, we propose selecting certain features of the information out to be conveyed via existing somatosensory information streams in native arms. In the native arm, haptic information is conveyed via canonical mechanoreceptors of the skin and their slowly adapting (SA) and rapidly adapting (RA) afferent components [12]. These mechanoreceptors have unique responses to the same stimuli [12], acting as the first step of information filtering in natural somatosensory function. This initial selectivity of information could be replicated by filtering out information from contact events to closely replicate the selectivity in native somatosensory function.

To accomplish this, one approach would be to mimic the somatosensory afferents of the native arm. For providing comprehensive somatosensory information, this paper will focus on SA and RA information streams and their two subtypes. SA and RA refer to the response of the mechanoreceptor to a sustained stimulus; SA mechanoreceptor afferents maintain a high response throughout the stimulus duration, where RA mechanoreceptor afferents respond to changes in stimulus intensity. Furthermore, the SA and RA mechanoreceptors can be broken down into their respective subtypes based on their intrinsic response properties. The frequency range refers to the range of frequencies, to which the mechanoreceptor can respond. Sensitivity refers to the lowest amount of change in frequency that will cause a change in the firing of the mechanoreceptor, and the receptor field size refers to the area on the surface of the skin which one mechanoreceptor occupies [12, 13].  Table 1 compares the subtypes of each RA and SA mechanoreceptor by these properties.

Sensory information produced by the response to contact events could possibly be replicated by filtering out the appropriate information from the fewest amount of sensors needed to replicate tactile sensation in the fingers and hand. The electrical signals from these sensors could be filtered through appropriate frequency band selection via bandpass filters or low-pass filters (or equivalent filter designs), followed by the addition of processing to recreate the response properties of the native sensory afferent (e.g., SA versus RA responses) according to their native physiological properties listed in Table 1 [13].

**Figure 4:**
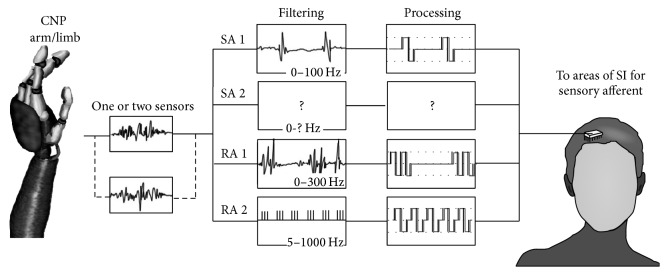
A schematic demonstrating possible filter designs and subsequent processing that could be used to replicate signals from native sensory afferents [13]. The proposed filters for band selection and processing are derived from the properties of sensory afferents from Table 1. SA I signals are shown being replicated by low-pass filtering to 100 Hz; then processing would be applied to replicate as close to 5 Hz steps of sensitivity applicable. RA I shows filtering followed by processing to replicate rapid adaptation as well as the sensitivity levels. RA II signals are shown being replicated by a hypothetical wavelet transform, showing the coefficients for a particular frequency range represented by a wavelet at a particular resolution. Since not much is known about the particular frequency range of SA II signals, it is listed blank. All filter designs and processing methods shown in the figure are hypothetical.

The processed signals must be used to evoke sensory percepts via ICMS to their relevant regions of S1 [13].  Figure 4 shows a proposed schematic of how the filtered sensory information from the sensor could be converted into ICMS pulse trains. A practical approach to replicating receptive field size would be to increase or decrease the number of sensors contributing to the ICMS for a particular column of S1 pertaining to the receptive field of a specific afferent system being simulated. In this process, the small receptive fields and fine spatial acuity of SA I responses are reproduced by selecting information from sensors that make up a low area and passing it through a low-pass filter 0–100 Hertz. Likewise, the large receptive field and spread spatial acuity of vibration sensitive RA II afferents could be replicated by allowing relevant higher frequency (5–1000 Hertz) information to band-pass filters from more sensors. RA I afferents would follow the same pattern as the previously mentioned signal breakdown techniques [13]. A method for reproducing SA II afferent responses is not proposed because not much is understood about the response properties of these mechanoreceptors.

ICMS pulse trains from the proposed method of signal processing could then be delivered to the respective areas of S1 for each receptive field of the hand and separated by their RA and/or SA stream components into the areas with these natural receptive fields. We hypothesize that using ICMS to deliver the same information processed by this method may improve the ability of a patient to interpret many details from elicited tactile percepts due to the existence of both RA and SA-like neurons in S1 [5].

The goal of this perspective is to propose a technique that could convey multiple submodalities of somatosensory stimuli to a human patient operating a somatosensory BMI. This point of view could offer insight into the recovery of multiple submodalities of tactile information as well as a deeper insight into the relationship between natural cortical coding of somatosensory information and the somatosensory percepts that they elicit.

## 6. Conclusion

Providing direct sensory feedback to amputees can be considered truly cutting edge research. A review of BMI and the superior control attained when incorporating somatosensory feedback into a device shows the possibility to restore natural function and the challenges that need to be addressed for this technology to reach a broad clinical setting. This paper offers a new perspective for improving tactile sensation in neural prosthetics with somatosensory feedback by separating the stimulus from the sensor into signals that resemble the sensory afferent sensation of a real hand. ICMS was discussed as a primary method for sending signals to the S1 in the postcentral gyrus of the brain. The applications of a feedback system range from reducing the cognitive burden of one sense to restoring vital sensations that are essential in allowing people the chance to interact with their surroundings and one another. The power that tactile sensation conveys is invaluable and completely necessary for restoring function. Moreover, it is certainly clear that research into creating prosthetic devices with sensory feedback will continue to evolve the scientific frontier, creating a new understanding of how the brain works and helping those suffering from the loss of limb use everywhere.

## Figures and Tables

**Table 1:** Skin in the native hand contains multiple types of low-threshold mechanoreceptors that contribute to tactile sensation. This table lists physiological characteristics and response properties from four canonical sensory mechanoreceptor afferents that could be utilized to process signals for sensory feedback. ^a^The optimal stimulus is the stimulus shown to result in the response shown. ^a,b^Table values adapted from [12, 13].

Receptor	Optimal stimulus^a^	Response properties^a^	Frequency range^b^	Sensitivity^b^	Receptor field size^b^
SA I Merkel disk	Indentation, points, curvature	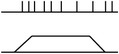	0–100 Hz	5 Hz	9 mm^2^

SA II Ruffini ending	Skin stretch, hand proprioception	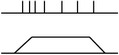	0-? Hz	0.5 Hz	60 mm^2^

RA I Meissner corpuscle	Skin movement	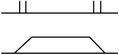	1–300 Hz	50 Hz	22 mm^2^

RA II Pacinian corpuscle	Vibration	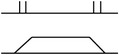	5–1000 Hz	200 Hz	Entire hand/finger
